# Fimasartan versus perindopril with and without diuretics in the treatment of elderly patients with essential hypertension (Fimasartan in the Senior Subjects (FITNESS)): study protocol for a randomized controlled trial

**DOI:** 10.1186/s13063-019-3466-5

**Published:** 2019-07-01

**Authors:** Min-gu Kang, Kwang-il Kim, Sang Hyun Ihm, Moo-Yong Rhee, Il Suk Sohn, Hae-Young Lee, Sungha Park, Eun-Seok Jeon, Jong-Min Song, Wook Bum Pyun, Ki-Chul Sung, Moo Hyun Kim, Sang-Hyun Kim, Seok-Yeon Kim, Shin-Jae Kim, Eung Ju Kim, Jinho Shin, Sung Yun Lee, Kook-Jin Chun, Jin-Ok Jeong, Shung Chull Chae, Ki Dong Yoo, Young Jin Choi, Yong Hwan Park, Cheol-Ho Kim

**Affiliations:** 1 Department of Internal Medicine, Chonnam National University Bitgoeul Hospital, Gwang-ju, 61748 Republic of Korea; 2 Department of Internal Medicine, Seoul National University College of Medicine, Seoul National University Bundang Hospital, Gumi-ro 166, Bundang-gu, Seongnam-si, Kyeongi-do 463-707 Republic of Korea; 3 Department of Internal Medicine, The Catholic University of Korea Bucheon ST. Mary’s Hospital, 327, Sosa-ro, Bucheon-si, Gyeonggi-do Republic of Korea; 4 Cardiovascular Center, Dongguk University Ilsan Hospital, 27, Dongguk-ro, Ilsandong-gu, Goyang-si, Gyeonggi-do Republic of Korea; 5 Department of Cardiology, Kyung Hee University Hospital at Gangdong, 892, Dongnam-ro, Gangdong-gu, Seoul, Republic of Korea; 6 Department of Internal Medicine, Seoul National University Hospital, 101 Daehak-ro, Jongno-gu, Seoul, Republic of Korea; 7 Department of Internal Medicine, Yonsei University Health System, Severance Hospital, 50-1, Yonsei-ro, Seodaemun-gu, Seoul, Republic of Korea; 8 Department of Internal Medicine, Samsung Medical Center, Sungkyunkwan, University School of Medicine, 81 Irwon-Ro Gangnam-gu, Seoul, Republic of Korea; 9 Department of Internal Medicine, Asan Medical Center, University of Ulsan College of Medicine, 88, Olympic-ro 43-gil, Songpa-gu, Seoul, Republic of Korea; 10 Department of Internal Medicine, Ewha Womans University Seoul Hospital, 260, Gonghangdae-ro, Gangseo-gu, Seoul, Republic of Korea; 11 Division of Cardiology, Department of Internal Medicine, Kangbuk Samsung Hospital, Sungkyunkwan University School of Medicine, 29, Saemunan-ro, Jongno-gu, Seoul, Republic of Korea; 12 Department of Internal Medicine, Dong-A University Hospital, 26, Daesingongwon-ro, Seo-gu, Busan, Republic of Korea; 13 Department of Internal Medicine, Seoul National University Borame Medical Center, 20, Boramae-ro 5-gil, Dongjak-gu, Seoul, Republic of Korea; 14 Department of Internal Medicine, Seoul Medical Center, 156, Sinnae-ro, Jungnang-gu, Seoul, Republic of Korea; 15 Department of Internal Medicine, Ulsan University Hospital, 877, Bangeojinsunhwando-ro, Dong-gu, Ulsan, Republic of Korea; 16 Department of Internal Medicine, Korea University Guro Hospital, 148, Gurodong-ro, Guro-gu, Seoul, Republic of Korea; 17 Department of Internal Medicine, Hanyang University Hospital, Wangsimni-ro, Seongdong-gu, Seoul, Republic of Korea; 18 Department of Internal Medicine, Inje University Ilsan Paik Hospital, 170, Juhwa-ro, Ilsanseo-gu, Goyang-si, Gyeonggi-do Republic of Korea; 19 Department of Internal Medicine, Pusan National University Yangsan Hospital, 20, Geumo-ro, Mulgeum-eup, Yangsan-si, Gyeongsangnam-do Republic of Korea; 20 Department of Internal Medicine, Chungnam National University Hospital, 282, Munhwa-ro, Jung-gu, Daejeon, Republic of Korea; 21 Chungnam National University, 99 Daehak-ro, Yuseong-gu, Daejeon, 34134 Republic of Korea; 22 Department of Internal Medicine, School of Medicine, Kyungpook National University, 130, Dongdeok-ro, Jung-gu, Daegu, Republic of Korea; 23 Department of Internal Medicine, The Catholic University of Korea, ST. Vincent’s Hospital, 93-1, Jungbu-daero, Paldal-gu, Suwon-si, Gyeonggi-do Republic of Korea; 24 Department of Internal Medicine, Sejong Hospital, 28, Hohyeon-ro 489beon-gil, Bucheon-si, Gyeonggi-do Republic of Korea; 25 Department of Internal Medicine, Samsung Changwon Hospital, Sungkyunkwan University School of Medicine, 158, Paryong-ro, Masanhoewon-gu, Changwon-si, Gyeongsangnam-do Republic of Korea

**Keywords:** Fimasartan, Perindopril, Hypertension, Essential hypertension, Treatment, Elderly, Frail elderly

## Abstract

**Background:**

Hypertension is an important risk factor for cardiovascular disease, even in the elderly. Fimasartan is a new non-peptide angiotensin II receptor blocker with a selective type I receptor blocking effect. The objective of this study is to confirm the safety and the non-inferiority of the blood pressure–lowering effect of fimasartan compared with those of perindopril, which has been proven safe and effective in elderly patients with hypertension.

**Methods:**

This is a randomized, double-blind, active-controlled, two-parallel group, optional-titration, multicenter, phase 3 study comparing the efficacy and safety of fimasartan and perindopril arginine. The study population consists of individuals 70 years old or older with essential hypertension. The primary outcome will be a change in sitting systolic blood pressure from baseline after the administration of the investigational product for 8 weeks. The secondary outcomes will be a change in sitting diastolic blood pressure from baseline and changes in sitting systolic blood pressure and diastolic blood pressure from baseline after the administration of the investigational product for 4, 16, and 24 weeks. The sample size will be 119 subjects for each group to confer enough power to test for the primary outcome.

**Discussion:**

Research to confirm the efficacy and safety of a new medicine compared with those of previously proven anti-hypertensive drugs is beneficial to guide physicians in the selection of therapeutic agents. If it is confirmed that the new drug is not inferior to the existing drug, the drug will be considered as an option in the treatment of hypertension in elderly patients.

**Trial registration:**

ClinicalTrials.gov Identifier: NCT03246555, registered on July 25, 2017.

**Electronic supplementary material:**

The online version of this article (10.1186/s13063-019-3466-5) contains supplementary material, which is available to authorized users.

## Background

Hypertension is a major risk factor for cardiovascular disease and affects over 1 billion people worldwide. From 1998 to 2014, the prevalence of hypertension in the population older than 65 years was 55.3–60.5% in Korea; thus, over half of the elderly individuals have hypertension. It is important to note that hypertension treatment can lower the risk of stroke, cardiovascular disease, and mortality [1, 2]. When a stroke or cardiovascular disease occurs, the quality of life and the daily life of the elderly are greatly affected. Therefore, there is an urgent need to study the treatment of hypertension in elderly individuals.

The Eighth Report of the Joint National Committee on the Prevention, Detection, Evaluation, and Treatment of High Blood Pressure aims to achieve a systolic blood pressure (SBP) of less than 150 mm Hg and a diastolic blood pressure (DBP) of less than 90 mm Hg in people older than 60 years [3]. There are differences among the guidelines, but the SBP target, in general, is set at less than 140–150 mm Hg. Angiotensin-converting enzyme (ACE) inhibitors, angiotensin II receptor blockers (ARBs), calcium channel blockers, and diuretics are recommended as first-line treatments for essential hypertension in elderly individuals without comorbidities. Among these, ACE inhibitors and ARBs have been proven to be clinically effective in the treatment of hypertension, as well as heart failure, by acting on the renin–angiotensin system, which acts as a main cause mechanism in cardiovascular disease [4].

The objective of this study is to confirm the safety and the non-inferiority of the blood pressure–lowering effect of fimasartan compared with those of perindopril in elderly individuals with essential hypertension. Perindopril arginine, selected as a control drug, is an ACE inhibitor used in the Hypertension in the Very Elderly Trial [2], a representative clinical study evaluating the effects of hypertension treatment in elderly patients older than 80 years. In the study, the mean blood pressure while sitting was 15.0/6.1 mm Hg lower in the active-treatment group than in the placebo group and fewer serious adverse events were reported in the active-treatment group. Based on these findings, perindopril was considered to be safe and effective when administered to elderly individuals. Fimasartan is a new non-peptide ARB with selective type I receptor blocking effect, which has been proven to be clinically effective in many randomized controlled trials [5, 6]. However, there are no clinical trials evaluating the efficacy and safety of this drug in patients over 70 years old. The response of elderly patients to medication may differ from that of the adult patient because the physiological changes due to aging affect the absorption, distribution, metabolism, and elimination of drugs [7]. As a result, the efficacy and safety of the drug may be different. For example, a study on risedronate has shown that risedronate has no significant effect on preventing hip fractures in women over 80 years of age [8]. Therefore, we would like to confirm the efficacy and safety of fimarsartan in elderly patients through a randomized, double-blind, multicenter trial.

Older patients are highly heterogeneous, and physiological ability and vulnerability vary widely, even for individuals of the same age [9]. Frailty, as a reflection of decreased physiologic reserve, is closely associated with biological age [10], concurrent medical conditions, morbidity, and decreased survival in elderly individuals [11]. Frailty assessments are clinically useful to address the heterogeneity of the health status among elderly individuals. The K-FRAIL test [12] and gait speed measurement [13], which are known to be useful methods for screening the frailty status, will be performed on the subjects, considering that the blood pressure–lowering effect of the anti-hypertensive drug may be different according to the frailty status. Using these results, we will analyze whether there is any difference in blood pressure–lowering effect according to the frailty status.

## Methods

### Study design

**Fig. 1:**
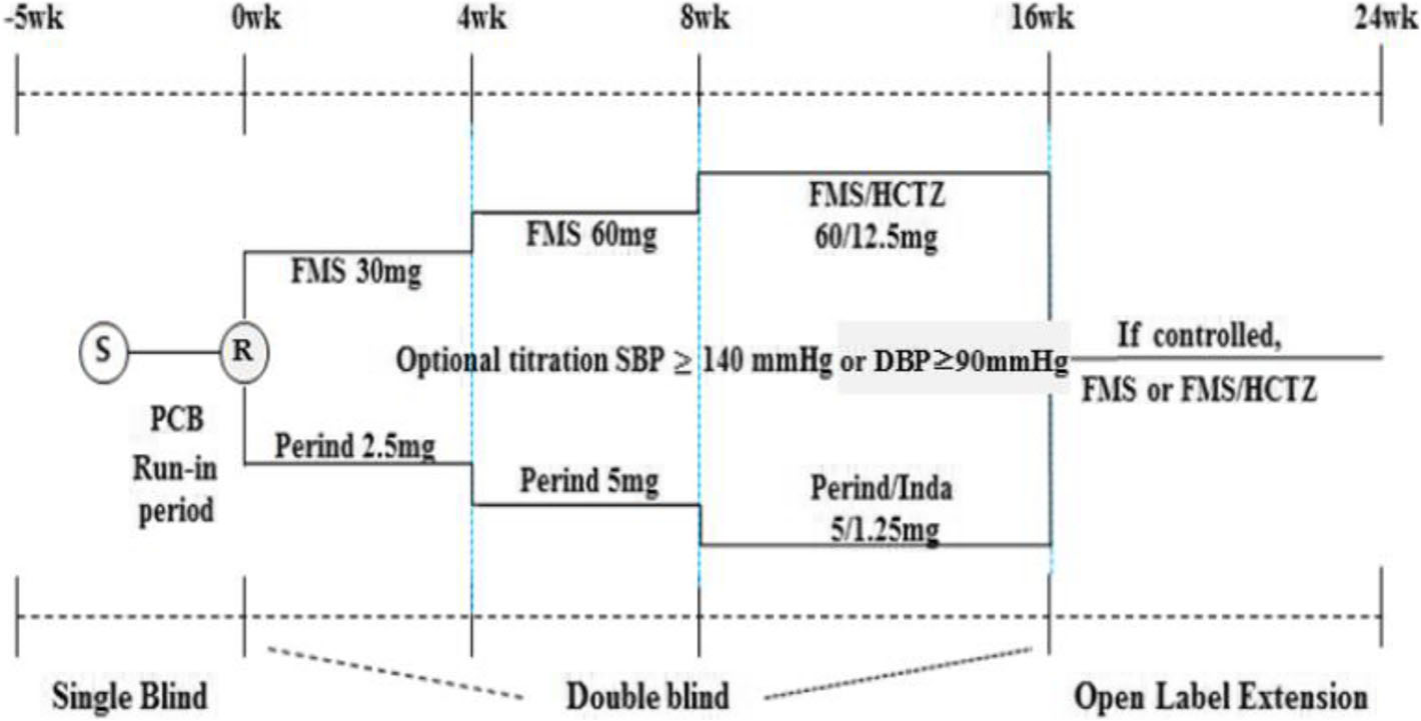
Study overview. *Abbreviations*: *FMS* Fimasartan, *HCTZ* Hydrochlorothiazide, *PCB* Placebo, *Perind* Perindopril arginine, *Inda* Indapamide, *R* Randomization, *S* Screening

**Fig. 2:**
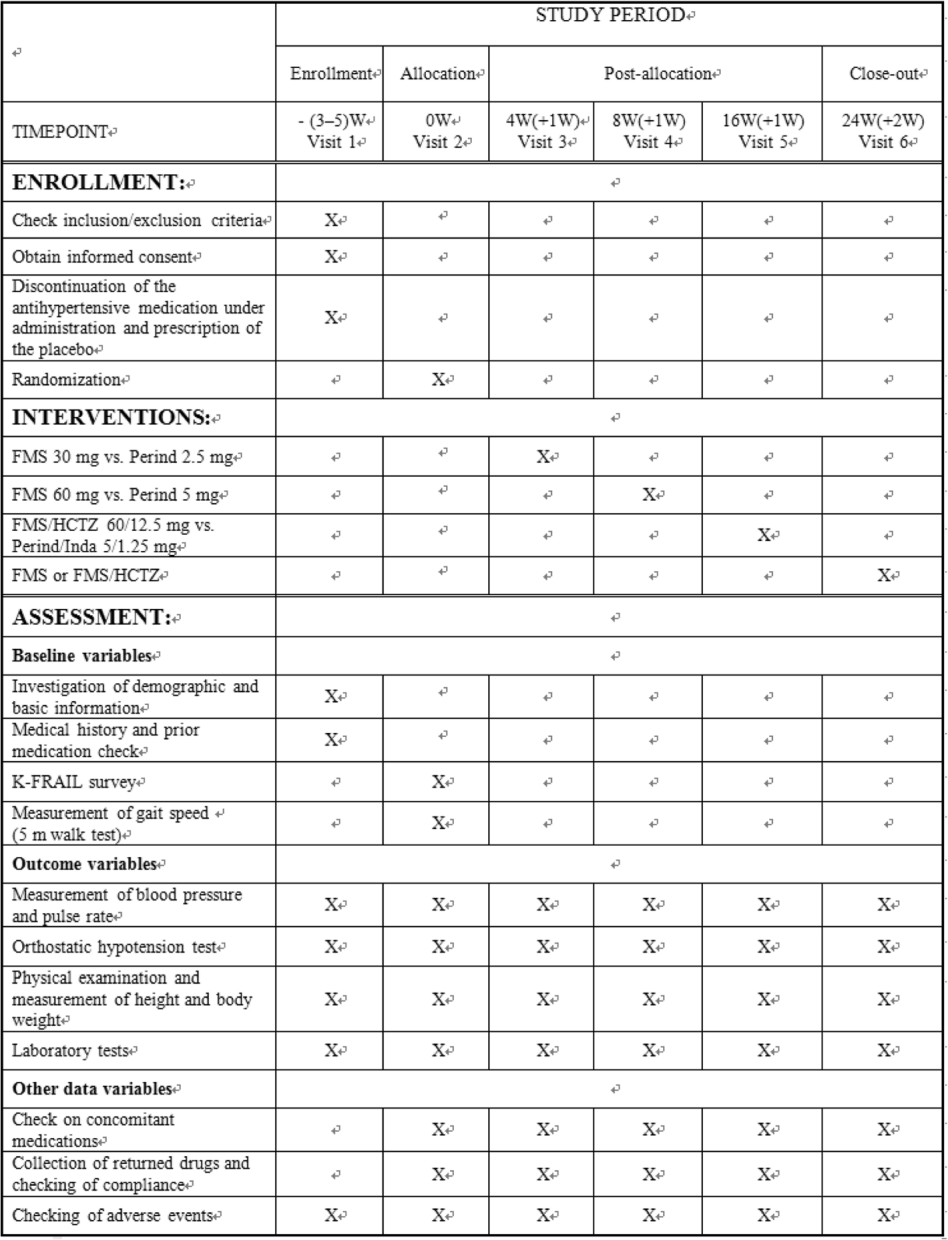
Time line of study procedure and outcome assessment. *Abbreviations*: *FMS* Fimasartan, *HCTZ* Hydrochlorothiazide, *Inda* Indapamide, *Perind* Perindopril arginine

This is a randomized, double-blind, active-controlled, two-parallel group, optional-titration, multicenter, phase 3 study designed to evaluate the blood pressure–lowering effect and safety of fimasartan and perindopril in elderly individuals with essential hypertension. The flow chart below captures the main events during the study (Fig. 1), and the time line of the study/outcome assessments is outlined in Fig. 2.

### Study population

#### Elderly with essential hypertension

Inclusion criteria are as follows:

1. Voluntarily provided written consent to participate in this clinical study after receiving an explanation of this study
2. Elderly (70 years old or older)
3. At the screening visit (Visit 1),

- Blood pressure: The mean blood pressure measured three times on the selected arm, as below:
- For treatment-naïve patients who have not taken drugs for hypertension within the last 3 months from the screening visit: mean siSBP (sitting systolic blood pressure) of at least 140 mm Hg
- For patients with essential hypertension who are taking drugs for hypertension: mean siSBP of at least 130 mm Hg

4) At the baseline visit (Visit 2),

- Blood pressure: Patients with mild to moderate essential hypertension whose mean siSBP measured three times on the selected arm is at least 140 mm Hg
- Patients with treatment compliance of at least 70% during the placebo run-in period

5) Capable of understanding written instructions, cooperative, and able to participate until the end of the clinical study.

Exclusion criteria are as follows:

1. Severe hypertension with mean siSBP of at least 180 mm Hg or siDBP (sitting diastolic blood pressure) of at least 110 mm Hg (office blood pressure) at the screening visit (Visit 1) and baseline visit (Visit 2). (However, at screening, it is based on the blood pressures measured from both arms, and the patient is excluded if the result from either of the arms falls within the criteria.)
2. Patients with a difference in siSBP of at least 20 mm Hg and siDBP of at least 10 mm Hg in the selected arm at the screening visit (Visit 1)
3. Patients with a history of secondary hypertension and any history of suspected secondary hypertension but not limited to the following conditions: coarctation of the aorta, primary hyperaldosteronism, renal artery stenosis, Cushing’s syndrome, pheochromocytoma, or polycystic kidney disease
4. Orthostatic hypotension with symptoms
5. Patients with insulin-dependent diabetes mellitus or uncontrolled diabetes mellitus (hemoglobin A1c [HbA1c] of more than 9.0% at the screening visit; Visit 1)
6. Patients with a history of malignant tumor, including leukemia and lymphoma, within the past 5 years (however, participation is allowed if it has not recurred for at least 5 years after a tumor surgery)
7. Patients with any chronic inflammatory disease requiring chronic anti-inflammatory treatment, consumption disease, autoimmune disease such as rheumatoid arthritis and systemic lupus erythematosus, or connective tissue disease at present or in the past
8. Patients with a history of a hypersensitivity reaction to any component of the investigational product and similar compounds: renin–angiotensin system inhibitors, ACE inhibitors, thiazide diuretics and sulfonamides, Yellow 5 (Sunset Yellow FCF (dye)), etc.
9. Patients with hyperlipidemia undergoing low-density lipoprotein (LDL) apheresis (patients undergoing LDL hemapheresis using a dextran sulfate cellulose)
10. Patients on dialysis, post-renal transplantation patients, or patients with clinically significant renal and hepatic diseases, such as cirrhosis, biliary tract obstruction, cholestasis, liver failure, and hepatic encephalopathy, or the following findings from tests at screening (Visit 1):

- Creatinine clearance (the modification of diet in renal disease (MDRD) equation) of less than 30 mL/min
- Alanine transaminase (ALT) or aspartate transaminase (AST) of at least three times the upper limit of normal (ULN)
- Hypokalemia (<3.5 mmol/L), hyponatremia (<133 mmol/L), or hyperkalemia (>5.5 mmol/L)

11) Patients with diagnosis of any of the following diseases or treatment for the following diseases within 6 months from the date of screening visit, which is deemed to be clinically significant by the investigator:

- Severe heart diseases: heart failure (New York Heart Association (NYHA) class III and IV), ischemic heart disease (angina pectoris or myocardial infarction), peripheral vascular disease, percutaneous transluminal coronary angioplasty, or coronary artery bypass grafting
- Hypertrophic obstructive cardiomyopathy, severe obstructive coronary artery disease, aortic stenosis, or hemodynamically significant aortic or mitral stenosis
- Clinically significant ventricular tachycardia, atrial fibrillation, atrial flutter, or arrhythmia
- Severe cerebrovascular disease (stroke, cerebral infarction, or cerebral hemorrhage)
- Known moderate or malignant retinopathy, such as retinal bleeding, visual disturbance, and retinal microaneurysm

12) Patients with angioedema caused by drugs, including ACE inhibitors, hereditary or idiopathic angioedema, or a history of such diseases
13) Patients with a history of alcohol or drug abuse within the last 2 years
14) Surgical or medical diseases that may affect the absorption, distribution, metabolism, and excretion of drugs that fall under one of the following cases (but not limited to them): history of major gastrointestinal surgery, such as gastrectomy, gastroenterostomy or small bowel resection, gastrointestinal bypass and gastrointestinal stapling, active gastritis, gastrointestinal or rectal bleeding at present, and active inflammatory bowel syndrome within the last 12 months
15) Patients with volume depletion, anuria, and shock
16) Patients with hereditary disorders of galactose intolerance, Lapp lactase deficiency, or glucose–galactose malabsorption
17) Patients who used other investigational products within 12 weeks prior to screening
18) Patients with any other reason that, in the opinion of the investigator, may contraindicate participation in the study.

### Study interventions

The treatment in this clinical study starts with fimasartan 30 mg or perindopril arginine 2.5 mg; if blood pressure is not controlled (siSBP of at least 140 mm Hg or siDBP of at least 90 mm Hg), the dose is escalated once gradually and then a diuretic combination is administered. The escalation of dose and administration of diuretics are decided after checking blood pressure 4 weeks after the initiation of each investigational product.

### Screening/placebo run-in period (single-blind)

If a subject voluntarily consents to participate in the study, the investigator will perform screening tests and a medical history survey to assess the subject’s eligibility. As a result of the screening tests, all subjects who are in compliance with the inclusion criteria and do not fulfill the exclusion criteria will have a placebo run-in period of 3–5 weeks and those who are on anti-hypertensive medications will stop the existing anti-hypertensive medication and take the placebo. The placebo run-in period proceeds on a single-blind basis.

### Dose titration period (double-blind)

After the completion of the placebo run-in period, assessments are performed to select the final subjects at the baseline visit, and the eligible subjects are randomly assigned to the fimasartan 30 mg group or the perindopril arginine 2.5 mg group at a 1:1 ratio. The randomly assigned subjects take two tablets of the relevant investigational product for each treatment group once daily for 4 weeks; in the case of patients with uncontrolled blood pressure (siSBP of at least 140 mm Hg or siDBP of at least 90 mm Hg) after 4 weeks, the dose is doubled (fimasartan 60 mg or perindopril arginine 5 mg) through an optional titration. The subjects take the investigational product at the escalated dose once daily for 4 weeks, and the patients still with uncontrolled blood pressure after the 4-week administration of the escalated dose take a diuretic combination (fimasartan 60 mg/hydrochlorothiazide 12.5 mg or perindopril arginine 5 mg/indapamide 1.25 mg) additionally. After 8 weeks, changes in their blood pressure are checked.

### Extension period (open-label)

The patients with controlled blood pressure (siSBP of less than 140 mm Hg and siDBP of less than 90 mm Hg) at week 16 participate in the 8-week open-label extension study after the dose for the test group is switched back to the previous dose and the dose for the control group is switched to the dose for the test group, and the efficacy and safety of the drugs are evaluated.

In the course of the clinical study, the subjects will make visits, including the screening and baseline visits, at weeks 4, 8, and 16 as well as at week 24 during the extension study (in the case of patients with controlled blood pressure at week 16), and they will undergo tests and assessments specified in the protocol at each visit.

### Study outcomes

The primary outcome will be a change in siSBP from baseline after the administration of the investigational product for 8 weeks.

The secondary outcomes will be as follows:

1. A change in siSBP from baseline after the administration of the investigational product for 4, 16, and 24 weeks
2. A change in siDBP from baseline after the administration of the investigational product for 4, 8, 16, and 24 weeks
3. Blood pressure response rate (siSBP of less than 140 mm Hg or decrease of siSBP of at least 20 mm Hg after the administration for 4, 8, and 16 weeks compared with baseline) and blood pressure normalization rate after the administration of the investigational products for 4, 8, and 16 weeks (siSBP of less than 140 mm Hg and siDBP of less than 90 mm Hg)
4. A change from baseline in the differences in standing SBP and DBP compared with their sitting measurements after the administration of the investigational product for 4, 8, 16, and 24 weeks.
5. Changes in siSBP and siDBP from baseline after the administration for 4, 8, 16, and 24 weeks depending on the level of senescence (healthy stage, pre-senescent stage, and senescent stage) evaluated by K-FRAIL
6. Changes in siSBP and siDBP from baseline after the administration for 4, 8, 16, and 24 weeks depending on gait speed (5-m walk test, <6 s, ≥6 s)

The change will be calculated by subtracting the baseline value from the value at the time of evaluation.

### Randomization

In this clinical trial, which is double-blinded, a randomization table is prepared by a stratified block randomization method where a study site is the stratification factor. For randomization, an independent statistician will generate randomization numbers by using SAS version 9.3 and will deliver them in an encrypted file to the director of investigational product packaging and IWRS (Interactive Web-based Response System) manager.

Subjects who meet all of the inclusion criteria and none of the exclusion criteria will be randomly assigned at a ratio of 1:1 to one of the following groups: treatment group (fimasartan 30 mg) or control group (perindopril 2.5 mg). Randomization will be carried out using IWRS.

For packaging and labeling of the investigational products, the sponsor’s investigational product packaging director will be in charge of properly packaging the study group and control group in accordance with the randomization table and labeling on the investigational product. The investigator will assign the randomization numbers through IWRS to the eligible subjects on the basis of the inclusion/exclusion criteria in the order of enrollment in the study and will prescribe the applicable investigational products. When dispensing the investigational products, the clinical trial pharmacist will reconfirm whether the subject’s randomization number matches the number labeled on the investigational product.

### Blinding

In the placebo run-in period and dose titration period of this study, a placebo with the same formulation and properties that is not distinguished by appearance is used to maintain double blindness. The tester, test subjects, and monitor personnel are not informed about which group the test subjects are assigned to.

In an emergency situation that threatens the safety of the subject, the investigator should promptly notify the person in charge of unblinding from Boryung Pharmaceutical Co., Ltd. (Seoul, South Korea). The person in charge of unblinding should decide whether to unblind in consultation with the investigator and document the decision. After consulting with the client, the investigator accesses the webpage about random assignment. After inputting the required information, the investigator confirms the clinical trial drug information of the subject and documents the unblinding. However, subjects and monitor personnel are not informed about unblinding. When the trial is over and the database is confirmed to be complete and accurate, the data will be locked and the random assignment code information will be released after the unblinding procedure.

### Sample size and statistical analysis

#### Sample size

In existing non-inferiority clinical trials of anti-hypertensive agents comparing the lowering effect of siSBP, the non-inferiority margin (δ) is set to 5–6 mm Hg [14, 15]. In this study, the non-inferiority margin was set at 5 mm Hg. As the standard deviation was 12.52 to 12.53 mm Hg in the fimasartan clinical trial for adult patients, the standard deviation (σ) was assumed to be 13 mm Hg in this study. Given a unilateral significance level of 2.5%, a power of 80%, and 1:1 allocation, the following formula was used to calculate the number of test subjects:

$$ \mathrm{n}=\frac{{\left({Z}_{\alpha }+{Z}_{\beta}\right)}^2{\sigma}^2\left(1+\frac{1}{K}\right)}{{\left(\varepsilon -\delta \right)}^2} $$

$$ \upvarepsilon =\upmu -\upmu =0 $$

δ: non-inferiority margin (5 mm Hg)

K: test group and control group allocation ratio (1:1)

α = 0.025, β = 0.2

σ = 13 mm Hg.

The required number of subjects for each group, calculated from the above formula, is 107. In consideration of a 10% dropout rate, a total of 238 subjects, 119 from each group, will be registered for enrollment.

### Efficacy

In this clinical trial, the efficacy evaluation will analyze mainly the data by per-protocol. We will analyze the effect of the drug on the subjects who have completed the trial without any serious violation of the plan.

#### Primary efficacy evaluation

To test the difference between the test group and the control group, an analysis of covariance (ANCOVA) will be performed with the baseline values as covariates and the treatment groups as effects. Change in siSBP from baseline after the administration of the investigational product for 8 weeks will be analyzed, and the difference between the treatment groups will be calculated by subtracting the change in the perindopril group from the change in the fimasartan group. The fimasartan group is determined to be non-inferior to the perindopril arginine group if the lower limit of the two-sided 95% confidence interval corresponding to the least square means (LSM) difference between the treatment groups is greater than −5 mm Hg.

#### Secondary efficacy evaluation

To test the difference between the groups, continuous variables will be analyzed through an ANCOVA with the baseline values as covariates and the treatment groups as effects. In the case of categorical variables, the frequencies (N) and percentages (%) of each period and their two-sided 95% confidence intervals will be presented; for an intergroup difference, logistic regression analysis will be performed with the baseline values as covariates. For the analysis of the changes within the treatment groups at week 24, a one-sample *t* test or a Wilcoxon signed-rank test will be performed.

In the subgroup analysis, the subjects will be divided into three groups, such as healthy stage, pre-senescent stage, and senescent stage, depending on the level of senescence evaluated by K-FRAIL. The subjects will also be divided into two groups, such as normal gait speed group and slow gait speed group, depending on gait speed evaluated by the 5-m walk test. For each subgroup, the descriptive statistics for the baseline values and the measurements at 4, 8, 16, and 24 weeks are presented. At 4, 8, and 16 weeks after the administration of clinical trial drugs, the change will be analyzed through an ANCOVA with the baseline values as covariates and the treatment as effects. For the analysis of the changes within the treatment groups at 24 weeks, a one-sample *t* test or a Wilcoxon signed-rank test will be performed. Using these results, we will analyze whether there is any difference in blood pressure–lowering effect according to the frailty status.

### Safety

Adverse events are coded and organized by using the MedDRA (Medical Dictionary for Regulatory Activities; version 19.0 or later), and the proportion of the subjects who have experienced adverse events and the 95% confidence interval are presented by treatment group. All adverse events are organized according to severity, and investigational product-related adverse events and serious adverse events are summarized separately. The chi-squared test or the Fisher’s exact test will be used to determine whether there is any difference in the incidence of adverse events between the groups.

For continuous data, such as hematology and blood chemistry results, the descriptive statistics (mean, standard deviation, median, minimum, maximum, etc.) will be presented by treatment group and visit; for categorical data, such as urinalysis and physical examination, the frequency (N) and percentage (%) will be presented by category. With regard to the difference in the continuous data before and after administration of the investigational product, the difference among visits will be tested by performing a paired *t* test or the Wilcoxon’s signed-rank test within the treatment group. A two-sample *t* test and a Wilcoxon rank-sum test will be carried out to test the difference in the change after the administration between the treatment groups. For the categorical variables, McNemar’s test will be performed for the intragroup change.

### Blood pressure measurements

Electronic blood pressure monitors (Omron HEM-7080IC, Omron Corporation, Kyoto, Japan) are used to measure blood pressure, and all laboratories use the same product. The blood pressure of both arms is measured by a trained study coordinator at the screening visit, and the arm with the higher average SBP obtained by three measurements is selected as the reference arm.

Blood pressure of the upper arm of the subject is measured in the sitting position at each visit. After the subject has rested in a sitting position for at least 5 min in a chair that can support the back and the arm has been fixed to the heart level, the blood pressure is measured. Blood pressure should be measured three times at intervals of more than 2 min, and to measure blood pressure at the lowest drug concentration, blood pressure measurements should be performed in the morning, before dosing.

### Data collection and management

Study data are collected and managed via the Medidata Rave™ (Medidata Solutions, Inc., New York, NY, USA, http://www.mdsol.com) electronic data capture (EDC) system.

## Discussion

Anti-hypertensive agents are widely used because of the high prevalence of hypertension and the clear effects of treatment. There are various anti-hypertensive drugs, such as diuretics, ARBs, ACE inhibitors, beta-blockers, and calcium channel blockers. Among these, ARBs and ACE inhibitors are the primary anti-hypertensive agents that reduce the activity of the renin–angiotensin system. As a result, direct vasoconstriction is blocked, sodium retention is reduced, and blood pressure is lowered.

Important factors to consider when choosing an anti-hypertensive agent are blood pressure–lowering effects and side effects. Sufficient blood pressure–lowering effect is very important because strict control of blood pressure has the advantage of lowering the risk of developing cardiovascular disease and mortality [16]. It is also advantageous in that drug compliance can be increased if the blood pressure can be sufficiently lowered by only one medication. We should also look at the side effects of the drugs. ACE inhibitors have side effects of dry cough and angioedema related to the increase in bradykinin levels, and ACE inhibitors and ARBs inhibit the synthesis of aldosterone, resulting in side effects of hyperkalemia. Consideration should be given to the choice of medicine, in view of the inherent side effects of individual drugs.

Because the prevalence of hypertension increases with age, research to confirm the efficacy and safety of new medicines compared with previously proven anti-hypertensive drugs for elderly individuals is beneficial for physicians to select appropriate medications. In particular, confirming the efficacy and safety of ARBs, which are used as the first-line drug in hypertension treatment, may be very helpful in selecting the appropriate medication. Adding diuretics as a second agent to patients whose blood pressure is not controlled despite an increased ACE inhibitor or ARB dose is an effective way to control blood pressure [17]. In addition, more information on drug safety for elderly individuals can be obtained by administering fimasartan or a combination of fimasartan and hydrochlorothizide for 8 weeks to all subjects during the open-label extension period.

There is an epidemiological study showing the difference in the rate of hypertension control according to the frailty status [18]. Therefore, it is meaningful to check whether the blood pressure–lowering effect of each drug is different depending on the frailty status. If an anti-hypertensive drug is less effective among the frail elderly people, blood pressure needs to be lowered sufficiently to the target using methods such as increasing drug dosage or using a combination drug.

Hypertension is widespread among elderly individuals, and hypertension treatment is effective in reducing cardiovascular disease and mortality. It is clinically significant to perform randomized controlled trials to confirm the efficacy and safety of fimasartan, which can be used as a first-line drug for hypertension treatment in elderly individuals. If fimasartan is not inferior to an ACE inhibitor that has been proven effective and safe, the drug will be considered as an option in the treatment of hypertension in elderly patients (Additional file 1).

## Trial status

Recruitment of patients started on August 7, 2017, and 131 subjects (55%) were randomly assigned as of May 17, 2018. Protocol version number 4.1. Date: November 6, 2017.

## Additional file

Additional file 1: SPIRIT 2013 checklist: Recommended items to address in a clinical trial protocol and related documents. (DOC 124 kb)

## Data Availability

Data sharing is not applicable to this article, as no datasets were generated or analyzed during the study.

## Abbreviations

ACE: Angiotensin-converting enzyme
ANCOVA: Analysis of covariance
ARB: Angiotensin II receptor blocker
DBP: Diastolic blood pressure
IWRS: Interactive Web-based Response System
LDL: Low-density lipoprotein
SBP: Systolic blood pressure
siDBP: Sitting diastolic blood pressure
siSBP: Sitting systolic blood pressure

## References

[1] Beckett N, Peters R, Tuomilehto J, Swift C, Sever P, Potter J (2011). Immediate and late benefits of treating very elderly people with hypertension: results from active treatment extension to Hypertension in the Very Elderly randomised controlled trial. BMJ.

[2] Beckett NS, Peters R, Fletcher AE, Staessen JA, Liu L, Dumitrascu D (2008). Treatment of hypertension in patients 80 years of age or older. N Engl J Med..

[3] James PA, Oparil S, Carter BL, Cushman WC, Dennison-Himmelfarb C, Handler J (2014). 2014 evidence-based guideline for the management of high blood pressure in adults: report from the panel members appointed to the Eighth Joint National Committee (JNC 8). JAMA.

[4] Werner CM, Bohm M (2008). The therapeutic role of RAS blockade in chronic heart failure. Ther Adv Cardiovasc Dis..

[5] Lee SE, Kim YJ, Lee HY, Yang HM, Park CG, Kim JJ (2012). Efficacy and tolerability of fimasartan, a new angiotensin receptor blocker, compared with losartan (50/100 mg): a 12-week, phase III, multicenter, prospective, randomized, double-blind, parallel-group, dose escalation clinical trial with an optional 12-week extension phase in adult Korean patients with mild-to-moderate hypertension. Clin Ther..

[6] Youn JC, Ihm SH, Bae JH, Park SM, Jeon DW, Jung BC (2014). Efficacy and safety of 30-mg fimasartan for the treatment of patients with mild to moderate hypertension: an 8-week, multicenter, randomized, double-blind, phase III clinical study. Clin Ther..

[7] Mangoni AA, Jackson SH (2004). Age-related changes in pharmacokinetics and pharmacodynamics: basic principles and practical applications. Br J Clin Pharmacol..

[8] MR MC, Geusens P, Miller PD, Zippel H, Bensen WG, Roux C (2001). Effect of risedronate on the risk of hip fracture in elderly women. Hip Intervention Program Study Group. N Engl J Med.

[9] Muller M, Smulders YM, de Leeuw PW, Stehouwer CD (2014). Treatment of hypertension in the oldest old: a critical role for frailty?. Hypertension.

[10] Mitnitski A, Collerton J, Martin-Ruiz C, Jagger C, von Zglinicki T, Rockwood K (2015). Age-related frailty and its association with biological markers of ageing. BMC Med..

[11] Klein BE, Klein R, Knudtson MD, Lee KE (2005). Frailty, morbidity and survival. Arch Gerontol Geriatr..

[12] Jung HW, Yoo HJ, Park SY, Kim SW, Choi JY, Yoon SJ (2016). The Korean version of the FRAIL scale: clinical feasibility and validity of assessing the frailty status of Korean elderly. Korean J Intern Med..

[13] Schoon Y, Bongers K, Van Kempen J, Melis R, Olde Rikkert M (2014). Gait speed as a test for monitoring frailty in community-dwelling older people has the highest diagnostic value compared to step length and chair rise time. Eur J Phys Rehabil Med..

[14] Malacco E, Vari N, Capuano V, Spagnuolo V, Borgnino C, Palatini P (2003). A randomized, double-blind, active-controlled, parallel-group comparison of valsartan and amlodipine in the treatment of isolated systolic hypertension in elderly patients: the Val-Syst study. Clin Ther..

[15] Na JO, Seo HS, Choi CU, Lim HE, Kim JW, Kim EJ (2013). Results of a 14-Week, Multicenter, Prospective, Randomized, Open-Label, Noninferiority Clinical Trial Comparing the Antihypertensive Effect and Edema Incidence of Lacidipine and Amlodipine in Older Korean Patients with Mild-to-Moderate Hypertension. Curr Ther Res Clin Exp..

[16] Williamson JD, Supiano MA, Applegate WB, Berlowitz DR, Campbell RC, Chertow GM (2016). Intensive vs Standard Blood Pressure Control and Cardiovascular Disease Outcomes in Adults Aged >/=75 Years: A Randomized Clinical Trial. JAMA.

[17] Palatini P (2005). Combination therapy in the management of hypertension: focus on angiotensin receptor blockers combined with diuretics. J Clin Hypertens..

[18] Kang MG, Kim SW, Yoon SJ, Choi JY, Kim KI, Kim CH (2017). Association between Frailty and Hypertension Prevalence, Treatment, and Control in the Elderly Korean Population. Sci Rep..

